# Neural basis of motivational approach and withdrawal behaviors in neurodegenerative disease

**DOI:** 10.1002/brb3.350

**Published:** 2015-07-14

**Authors:** Shunichiro Shinagawa, Adhimoolam Babu, Virginia Sturm, Tal Shany-Ur, Parnian Toofanian Ross, Diana Zackey, Pardis Poorzand, Scott Grossman, Bruce L Miller, Katherine P Rankin

**Affiliations:** 1 Memory and Aging Center, Department of Neurology, University of CaliforniaSan Francisco, California; 2 Department of Psychiatry, Jikei University School of MedicineTokyo, Japan

**Keywords:** Alzheimer’s disease, behavior inhibition, dementia, frontotemporal dementia, neurodegeneration, social concern, voxel-based morphometry

## Abstract

**Introduction:**

The Behavioral Inhibition System (BIS) and the Behavioral Activation System (BAS) have been theorized as neural systems that regulate approach/withdrawal behaviors. Behavioral activation/inhibition balance may change in neurodegenerative disease based on underlying alterations in systems supporting motivation and approach/withdrawal behaviors, which may in turn be reflected in neuropsychiatric symptoms.

**Method:**

A total of 187 participants (31 patients diagnosed with behavioral variant of FTD [bvFTD], 13 semantic variant of primary progressive aphasia [svPPA], 14 right temporal variant FTD [rtFTD], 54 Alzheimer’s disease [AD], and 75 older healthy controls [NCs]) were included in this study. Changes in behavioral inhibition/activation were measured using the BIS/BAS scale. We analyzed the correlation between regional atrophy pattern and BIS/BAS score, using voxel-based morphometry (VBM).

**Results:**

ADs had significantly higher BIS scores than bvFTDs and NCs. bvFTDs activation-reward response (BAS-RR) was significantly lower than ADs and NCs, though their activation-drive (BAS-D) was significantly higher than in ADs. Both AD and rtFTD patients had abnormally low activation fun-seeking (BAS-FS) scores. BIS score correlated positively with right anterior cingulate and middle frontal gyrus volume, as well as volume in the right precentral gyrus and left insula/operculum.

**Conclusions:**

AD, bvFTD, and rtFTD patients show divergent patterns of change in approach/withdrawal reactivity. High BIS scores correlated with preservation of right-predominant structures involved in task control and self-protective avoidance of potentially negative reinforcers. Damage to these regions in bvFTD may create a punishment insensitivity that underlies patients’ lack of self-consciousness in social contexts.

## Introduction

There have been decades of research investigating the neural underpinnings of the motivational systems regulating approach and withdrawal behavior. One dominant theory proposed by Gray (1987), suggests that two general neural systems coordinate adaptive behavior, the behavioral inhibition system (BIS), and the behavioral activation system (BAS). They suggest that the BIS is involved in processing signals related to punishment, novelty, and fear. Its function is to increase attention toward fear stimuli, to interrupt ongoing behavior, and prepare for vigorous action. Individuals with high BIS measurements have greater physiological reactivity and report higher rates of negative emotions, in particular anxiety (Arnett and Newman 1999). The complementary BAS system is theorized to control approach behavior in response to reward cues and positive affect. This theory is one of the most dominant biologically based personality theories, and has motivated investigations of the neurophysiological (Coan and Allen 2003) (Hawk and Kowmas 2003; Reuter et al. 2006), and molecular genetic basis of behavioral inhibition and activation in multiple experimental contexts and patient populations (Cools et al. 2005). Theoretically, because approach and avoidant behaviors are often enacted in social exchanges, the opposing circuits underlying the BIS/BAS motivational theory likely also extend to interpersonal behavior, and may elucidate the neural basis for social drive.

Carver and White developed a questionnaire measure of individual differences in these behavioral inhibition/activation tendencies, the BIS/BAS scales (Carver and White 1994). Scores on these questionnaires have been shown to reflect individual differences in psychophysiological reactivity as well as level of functional activity of the brain structures underlying the behavioral inhibition/activation system, in the presence of situational cues of impending threat and reward. Heightened imbalance between the behavioral inhibition/activation system, as measured by the BIS/BAS scales, has been observed in various psychiatric disorders (Johnson et al. 2003). For instance, patients with schizophrenia show abnormally high sensitivity to threat on the BIS scale (Scholten et al. 2006); depression is associated with lower BAS levels and higher BIS levels (Kasch et al. 2002); bipolar disorder is associated with abnormal elevations in both BIS and BAS (Meyer et al. 2001); and patients with attention-deficit hyperactivity disorder (ADHD) show abnormally low BIS reactivity (Matthys et al. 1998). Given that neurodegenerative and psychiatric disorders often cause early circuit disruption in the same networks (Bora et al. 2010), similar imbalances between approach and withdrawal behaviors might also appear in neurodegenerative disease. For instance, although Alzheimer’s disease (AD) is not known for dramatic social behavior changes, personality changes such as decreased extraversion and increased anxiety and neuroticism do occur in the very early stages of AD (Duchek et al. 2007; Seignourel et al. 2008; Sollberger et al. 2009; Sturm et al. 2013), suggesting that heightened behavioral inhibition and motivated social withdrawal may be early signs of BIS/BAS circuit disruption. This is especially true in AD patients with more rapid cognitive decline and earlier age at onset (Porter et al. 2003). In contrast, drastic changes in personality and social behavior are core features in patients with behavioral variant frontotemporal dementia (bvFTD) (Rascovsky et al. 2011), and are the direct result of neurodegeneration (Snowden et al. 2001; O’Callaghan et al. 2013). In particular, bvFTD patients demonstrate a variable mix of two primary behavioral features that could be characterized as disruption in motivational systems: disinhibition and apathy. They are not sensitive to punishment cues in a social setting, and characteristically demonstrate a paradoxical mixture of diminished drive toward abstract, socially approved goals alongside increased drive toward shallow, stereotyped behavioral goals.

However, to date, neurodegenerative disease patients have not been characterized with respect to these systems regulating behavioral activation and inhibition, and the relationship of these systems to patients’ increased introversion, anxiety, disinhibition, and apathy remains unclear. In the present study, our purpose is (1) to identify whether neurodegenerative disease patients with bvFTD, semantic variant of primary progressive aphasia (svPPA), right temporal variant of FTD (rtFTD), and AD show changes in behavioral activation, inhibition as measured by the BIS/BAS scale; (2) to reveal the neural correlates of any abnormalities in behavioral activation/inhibition in these patients using voxel-based morphometry (VBM) of structural MRI; and (3) to demonstrate how changes in BIS/BAS system corresponds to more broadly defined neuropsychiatric symptoms in these patients.

## Method

### Participants

A total of 187 subjects were enrolled in this study, including healthy controls and patients recruited through the Memory and Aging Center at the University of California San Francisco. Patient diagnosis was derived by a multidisciplinary team consisting of neurologists, neuropsychologists, psychiatrists, and nurses, who performed extensive behavioral, neuropsychological, and neuroimaging assessments. Among them, 31 patients met the International bvFTD Criteria Consortium research diagnostic criteria for bvFTD (Rascovsky et al. 2011). Twenty-seven patients with predominantly temporal atrophy met international criteria for svPPA (Gorno-Tempini et al. 2011). Because of the heterogeneity of behavioral symptomatology within this diagnostic category (Rankin et al. 2003; Liu et al. 2004), these patients were further subdivided into two groups depending on clinical presentation. Patients with predominantly left temporal atrophy (svPPA) present with loss of semantic knowledge of words and objects, but have less severe behavioral and personality changes. On the other hand, patients with predominantly right temporal atrophy present with clear loss of empathy and ability to read social cues, more mental rigidity, and other behavioral symptoms that overlap with and often meet FTDC diagnostic criteria for bvFTD, while their language symptoms are subtle or absent (Seeley et al. 2005). Thus, in this study, 14 predominantly temporal patients with substantial semantic loss for object knowledge were categorized as svPPA, and 13 patients without substantial loss of object knowledge with predominantly behavioral presentation were classified as rtFTD (Seeley et al. 2005; Josephs et al. 2009). Patients with nonfluent variant primary progressive aphasia (nfvPPA) were excluded because too few were available over the course of enrollment to complete quantitative statistical analysis. In addition to these FTD groups, 54 subjects meeting current diagnostic criteria for probable AD with appropriate biomarker evidence (McKhann et al. 2011) and 75 older healthy controls (NCs) were also recruited.

All subjects underwent comprehensive neuropsychological battery including Mini Mental State Examination (MMSE), working memory task (digit span backwards), verbal episodic memory (California Verbal Learning Test), visual episodic memory (memory for modified Rey–Osterrieth figure), visual-spatial function (copy of a modified Rey–Osterrieth figure), confrontational naming (Boston Naming Test), sentence comprehension and repetition, phonemic, semantic, and nonverbal fluency, visual-motor sequencing (a modified version of the Trails B test), and so on (See Rosen et al. 2002 for more detail). All subjects were required to have an informant to corroborate their daily functioning. Informants were typically relatives who lived with the subject, and were required to have known the subject for more than 5 years. All the assessments including brain imaging were conducted within 6 months. The subjects and their informants signed an institutional review board-approved research consent form to participate in the study.

### Assessment of motivational systems

To assess participants’ characteristic functioning in the behavioral activation and behavioral inhibition systems described above, the standard BIS/BAS questionnaires (20 items on a 5-point scale) were administered to the informants, who were asked to describe the participant’s typical behavior (Carver and White 1994). The BIS Scale (seven items), measuring behaviors consistent with a withdrawal motivation, contains items describing the degree to which people characteristically express anxiety when confronted with cues for punishment. The BAS Scale (13 items) targets behaviors consistent with an approach motivation, and is divided into subscales representing reward responsiveness (BAS-RR: five items), drive toward appetitive goals (BAS-D: four items), and fun-seeking (BAS-FS: four items). Several large validation studies found support for the construct validity of the BIS/BAS scales, such as in an Australian community sample (*N* = 2725) (Campbell-Sills et al. 2004), and a sample of American outpatients with anxiety and mood disorders (*N* = 1825) (Jorm et al. 1999). Cultural generalizability has been demonstrated across samples from the USA, UK, and Italy (*N* = 646) (Scholten et al. 2006).

### Neuropsychiatric assessment

Subjects were also evaluated with the Geriatric Depression Scale (GDS), a 30-item self-report questionnaire (Yesavage et al. 1982). Behavior was also measured using the Neuropsychiatric Inventory (NPI), a caregiver interview designed to assess the frequency and severity of behaviors that commonly occur as a result of a dementia syndrome (Cummings 1997).

### Structural MRI

Participants underwent 1.5T (*N* = 12), 3T (*N* = 151), or 4T (*N* = 24) research quality structural MRI within 5 months of completing the BIS/BAS scale. Scanner heterogeneity occurred due to the time course of clinical research data collection for this study. Images were acquired on a 1.5T Siemens Magnetom VISION system (Siemens, Iselin, NJ), equipped with a standard quadrature head coil, using a magnetization prepared rapid gradient echo (MPRAGE) sequence; on a 3.0 Tesla Siemens (Siemens) TIM Trio scanner equipped with a 12-channel head coil using volumetric MPRAGE; and on a 4T Bruker MedSpec system with an 8-channel head coil controlled by a Siemens Trio console, using an MPRAGE sequence.

### Voxel-based morphometry (VBM)

Structural T1 images were visually inspected for movement artifact; corrected for bias field; segmented into gray matter, white matter, and cerebrospinal fluid; and spatially normalized to MNI space, using the statistical parametric mapping (SPM8) software package *(Welcome Department of Cognitive Neurology, London;* http://www.fil.ion.ucl.ac.uk/spm*)*. The decision to combine all the three types of scans together for VBM analysis, explicitly entering scanner type as a nuisance covariate, was based on a number of factors. First, these are rare disease patients for which all available data should be used if possible to improve statistical power, as long as signal-to-noise ratios are likely to still yield meaningful results. Second, validation studies examining clinically relevant outcomes of structural neuroimaging VBM analyses using images of neurodegenerative disease patients collected across different modes of hardware have shown that the downstream effects of scanner type are minimal after brains are normalized to template, and thus are unlikely to cause artifacts that are potentially clinically meaningful at the level of strict statistical thresholds (Abdulkadir et al. 2011).

The diffeomorphic anatomical registration through exponentiated lie algebra (DARTEL) toolbox was used to warp each participant’s image to a template created from 144 comprehensively screened neurologically normal older adults (age 70.3 ± 7.4, range 48–89 years; 53% female) to optimize intersubject registration (Ashburner 2007). Gray matter maps were then smoothed with an 8-mm full-width at half-maximum Gaussian kernel. Default settings for SPM8 with DARTEL were used with the exception that light cleanup of voxels was performed.

### VBM analyses of behavioral inhibition and activation

Covariates-only (multiple regression design) statistical analyses were used to determine the relationship between BIS/BAS score and smoothed grey matter volumes across all subjects in the sample.

There were several reasons for including participants with FTD and AD together with NCs in the study. First, greater variance of behavioral activation/inhibition score and grey matter volume increased the statistical power to detect brain–behavior relationships across the whole brain in this primarily correlational design. Second, inclusion of NCs ensured that the normal end of the regression line was represented in all analyses, regardless of the brain region or behavior in question. Scatterplots of peak regions were reviewed to confirm uniform dispersion across the whole range regardless of participant group.

Age, gender, MMSE, type of MRI scans, and total intracranial volume (TIV) were entered as covariates into all designs. MMSE is a widely used test for screening cognitive impairment of patients with dementia, which is used as a proxy for disease severity among all disease groups in this study. The resulting statistical parametric maps (SPM) were thresholded voxelwise at *P *<* *0.001, and then corrected for family-wise error at *pFWE *< 0.05 based on cluster extent and a custom-fit error distribution determined by 1000 permutations of the data (Wilson et al. 2010). Permutation analysis is a resampling approach to significance testing by which a test statistic is compared with the null distribution derived from the present study’s data set, and thus yields an accurate, study-specific t-threshold for type 1 error at *P* < 0.05 across all voxels (Kimberg et al. 2007).

The following statistical analyses were performed:

1. *Main Effects Analysis (voxel-wise regression of grey matter volume on BIS/BAS score)*: To identify volumetric correlates of BIS/BAS, separate design matrixes were constructed for BIS and each of the three BAS subscale scores, using a one-tailed t-contrast, adjusting for age, gender, MMSE, type of MRI scan, and TIV [1 0 0 0 0 0].
2. *First Co-Atrophy Error Check*: Shared Effect Analysis (voxel-wise regression of grey matter volume on BIS/BAS score controlling for diagnostic group effects and amount of change).

Because the diagnosis-driven clustering of atrophy patterns inherent in our sample had the potential to lead to “co-atrophy” error, we performed an additional analysis to determine if the brain–behavior relationships observed in the main effect analyses held true in more than one diagnostic group (Sollberger et al. 2011). We binarized each diagnosis and entered all four groups into the design matrix as additional confounding covariates. The results of this analysis show regions of atrophy significantly related to the discrepancy score only if they appear in more than one diagnostic group, confirming the generalizability of any brain–behavior relationships detected in the main effect analysis. However, these results should not be considered independently from the main effects results, because this approach will fail to identify any brain region that is legitimately related to the BIS/BAS score but is atrophied only in a single diagnostic group. We accepted a level of significance of *P *<* *0.001 uncorrected within the brain areas of interest previously identified in the main effects analysis, and *pFWE *< 0.05 for areas outside of these regions of interest.

1. *Second Co-Atrophy Error Check*: Linear Regressionccc Comparison of Significant Peak Voxels (regression of BIS/BAS score on grey matter volumes at peak coordinates).

We further assessed for coatrophy error by performing an additional multivariate analysis of peak regions identified in the main effect analysis. We extracted voxel intensities for each subject at each peak coordinate from the smoothed grey matter images, then we performed a linear regression analysis of the voxel values using SAS 9.4 (SAS Institute, Cary, North Carolina), with all voxel values modeled together as predictors of BIS/BAS score, and included age, gender, MMSE, type of MRI scan, and TIV as potential confounds. Brain regions that remained significant predictors of BIS/BAS, despite being modeled together with other peak regions, were considered to independently predict BIS/BAS score, providing additional confirmation that their presence in the main effects results was not simply due to coatrophy bias.

## Results

### Demographic results

Table 2011 shows the characteristics of subjects classified by diagnostic group. An omnibus general linear model with an alpha level of *P* < 0.05 and post hoc pairwise comparisons to the control sample using a Dunnett-Hsu test were conducted. Differences in gender distribution across groups were compared by chi-square test, which resulted in no significant differences. AD, bvFTD, and svPPA groups were significantly younger than NC groups, thus age was included as a covariate in all analyses. AD and bvFTD groups were significantly lower in education than NCs, though the actual difference between means was only 1.5 years of education, suggesting that this statistical difference resulted from the extremely tight distribution of education in our controls, and may not have any clinical significance at the high level of education demonstrated in all groups. AD, bvFTD, and svPPA groups had significantly lower MMSE scores than the NC group. AD, bvFTD, svPPA, and rtFTD groups had significantly higher GDS scores than NCs. All subject groups were equally likely to have undergone MRI scanning on any of the three scanners (1.5T/3T/4T, n.s.), but scanner type was still included in all VBM analyses as a precaution. Table 2007 shows the characteristics of BIS/BAS score in NCs, divided into four age groups. There were no significant differences among groups in BIS or BAS scores across age groups, controlling for gender and education. We also performed a regression analysis to directly assess whether BIS/BAS scores change as a function of age in our NC group, controlling for gender and education, and age did not significantly predict BIS or BAS scores. Men and women did not significantly differ on any BIS/BAS score.

**Table 1:** Characteristics of subjects classified by diagnostic groups

	AD (*n* = 54)	bvFTD (*n* = 31)	rtFTD (*n* = 13)	svPPA (*n* = 14)	NC (*n* = 75)	*F*-value
Gender (M/F)	27/27	22/9	7/6	8/6	34/41	χ^2^ = 6.05
Age	63.46 (8.68)†	60.52 (9.47)†	63.92 (5.91)	61.07 (7.53)†	69.79 (8.18)	9.52*
Education	16.19 (2.59)†	15.33 (2.95)†	16.31 (3.25)	16.50 (2.50)	17.73 (2.19)	5.84*
MMSE	21.96 (5.95)†	23.58 (7.14)†	25.58 (5.56)	20.77 (7.01)†	29.30 (0.90)	24.18*
CDR (0.5/1/2/3)	27/23/3/0	2/15/10/4	8/4/1/0	7/6/1/0		χ^2^ = 34.48
GDS	7.49 (4.76)†	8.68 (6.53)†	7.15 (6.82)†	10.42 (5.48)†	2.34 (2.84)†	17.52*

* *P* < 0.05.

† *P* < 0.05 versus NCs based on post hoc Dunnett’s test.

AD, Alzheimer’s disease; bvFTD, behavioral variant frontotemporal dementia; svPPA, semantic variant of primary progressive aphasia; rtFTD, right temporal variant frontotemporal dementia; NC, normal control; MMSE, Mini Mental State Examination; CDR, Clinical Dementia Rating; GDS, Geriatric Depression Scale.

**Table 2:** Effect of normal aging on BIS/BAS score

Age group	46–64 (*n* = 17)	65–69 (*n* = 23)	70–74 (*n* = 17)	75–90 (*n* = 18)	*F* value
Gender (M:F)	5:12	8:15	12:5	9:9	χ^2^ = 7.31
BIS total	15.1 (2.4)	15.65 (3.6)	16.35 (3.6)	16.44 (3.7)	0.891
BAS-D	9.9 (2.9)	10.04 (2.3)	9.71 (2.6)	10.17 (2.2)	0.11
BAS-RR	15.3 (2.9)	15.83 (2.0)	14.35 (3.0)	14.39 (2.3)	1.62
BAS-FS	9.9 (3.6)	10.74 (2.1)	9.18 (2.0)	9.5 (1.4)	1.66

### Diagnostic group differences in BIS/BAS

**Figure 1:**
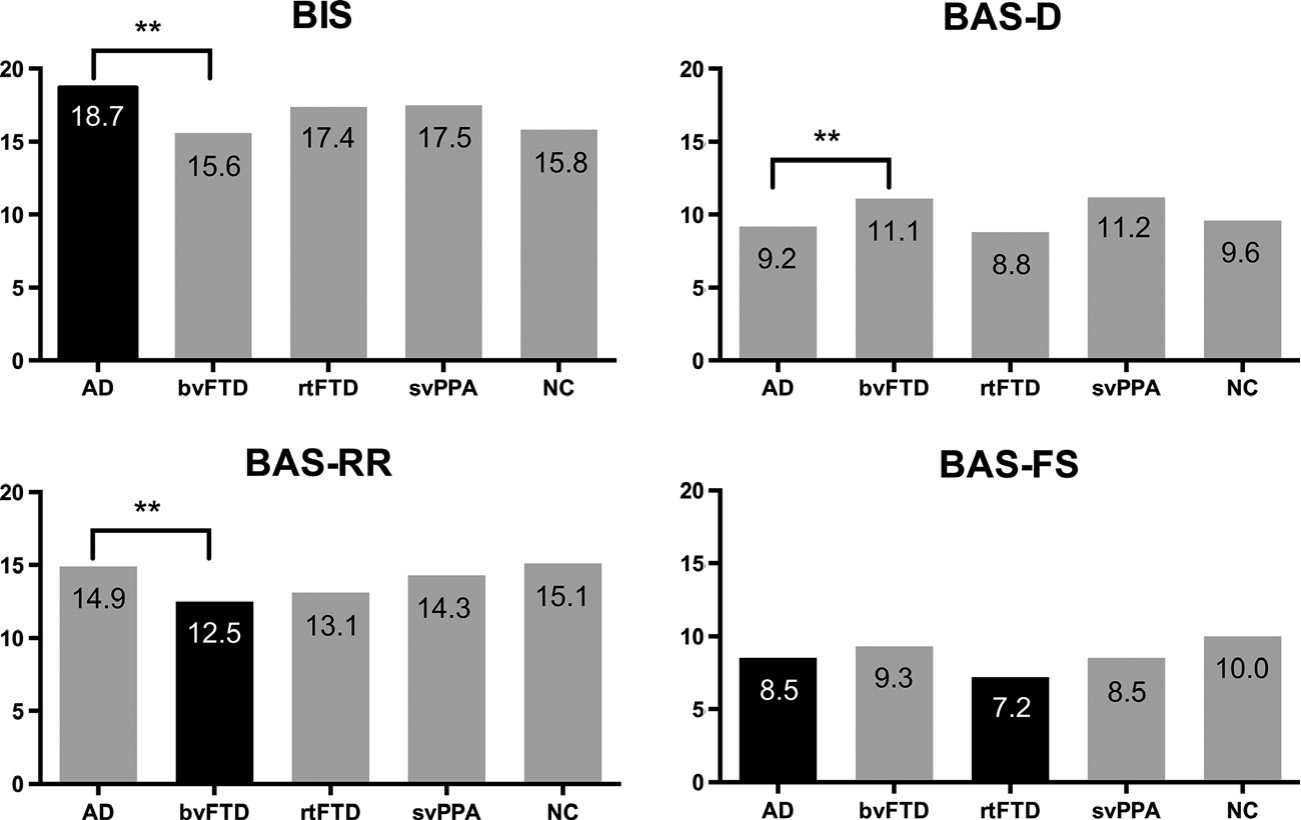
BIS/BAS score of each diagnostic groups. Significance is denoted by **P <* *0.01. Black bar denotes significant (*P *<* *0.01) difference from the normal control group.

Figure1 shows the BIS and BAS subscores in each diagnostic group. General linear models adjusted for age, gender, and MMSE score with an alpha level of <0.05 and post hoc pairwise comparisons among all groups were conducted using a Tukey–Kramer test. AD patients scored significantly higher in BIS score than bvFTD and NC groups (*P* < 0.01). bvFTD patients scored significantly higher in BAS-D score than AD patients (*P* < 0.05). bvFTD patients scored significantly lower in BAS-RR score than AD and NC groups (*P* < 0.01). AD (*P* < 0.05) and rtFTD (*P* < 0.01) patients scored significantly lower in BAS-FS score than NC subjects.

### Neural correlates of BIS score

Higher levels of BIS score correlated with reduced volume in the right anterior cingulate, right precentral gyrus, right middle frontal gyrus, right superior frontal gyrus, and left insula, (*pFWE *< 0.05; Table 1999, Fig.2). The right anterior cingulate result remained significant when diagnosis was included in the model, and peaks representing the right anterior cingulate, right precentral gyrus, and right middle frontal gyrus remained significant, independent predictors of BIS score when the five main effect peaks were modeled together in a multivariate regression (Table 1999).

**Table 3:** Neural substrates of BIS score

Anatomic region	mm^3^	*x*	*y*	*z*	*t*-value	*β*-weight
R anterior cingulate^1^	1801	14	39	0	3.93	0.21^2^
R precentral gyrus	408	58	3	15	4.11	0.18^2^
R middle frontal gyrus	402	28	63	−6	3.72	0.22^2^
R superior frontal gyrus	179	6	59	30	3.79	0.44
L insula	235	−44	8	3	3.79	0.34

Regions where BIS score negatively correlated with grey matter volume, adjusting for age, gender, MMSE, scanner type, and total intracranial volume (TIV) (corrected for family-wise error (FWE) across the whole brain at a significance level of *P *<* *0.05).

1 Region remains significant when diagnostic group is entered into the model.

2 Region remains a significant independent predictor of BIS score when all peaks modeled together in a regression analysis.

**Figure 2:**
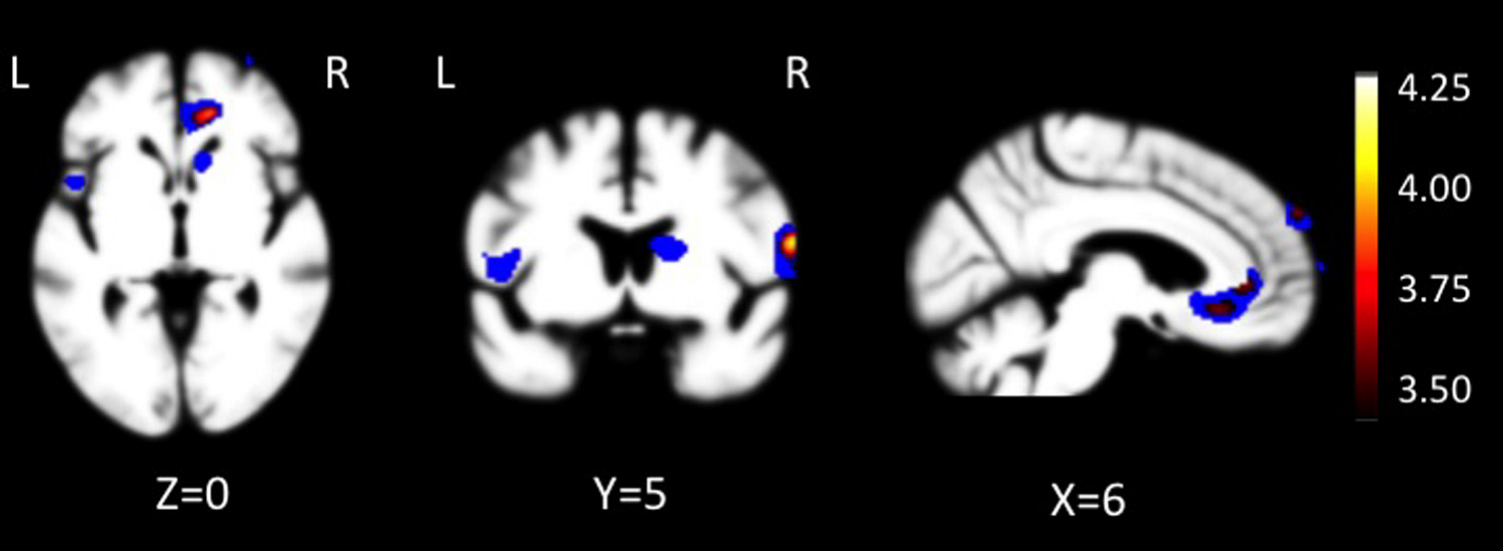
Neural substrates of BIS score. T-score maps of brain areas for which larger volume is associated with higher BIS score when controlling for age, education, gender, type of MRI scan, and total intracranial volume. Areas include right anterior cingulate, right precentral gyrus, right middle frontal gyrus, left dorsal anterior insula/operculum, and right superior frontal gyrus (pFWE < 0.05). Color bar represents T-scores (hot, *T* = 3.72, pFWE < 0.05 according to study-specific permutation analysis; blue, *P *<* *0.001, uncorrected; *T* > 3.18 and cluster size >150 mm^3^). Images were overlaid with MRIcron (http://www.mccauslandcenter.sc.edu/ CRNL/) on an average brain based on the healthy older control gray matter template used for DARTEL warping.

### Neural correlates of BAS-D, BAS-FS, BAS-RR subscores

None of the three subscores of the BAS scales were able to significantly predict grey matter volume in these patient groups after the analysis was corrected for family-wise error (*pFWE *< 0.05). Anatomic regions significant at an uncorrected (*P* < 0.001) threshold diverged significantly across subscales, with the dorsal anterior cingulate gyrus predicting drive (BAS-D), the dorsomedial frontal gyrus predicting fun-seeking (BAS-FS), and regions in the right subgenual cingulate predicting reward responsiveness (BAS-RR). (T-maps of these three BAS subscores can be viewed in Supplemental Figure S1). Because the BAS subscale scores yielded very anatomically divergent regions, this suggested that there was not a psychometrically valid reason to combine these diverse measures for a BAS-total anatomic analysis.

### BIS/BAS as predictors of neuropsychiatric symptoms

To examine whether BIS/BAS scores corresponded to other informant-based or self-report measures of neuropsychiatric symptoms, we examined partial correlations, controlling for gender and age, between BIS/BAS scores, GDS score (self-reported depressive symptoms), and overall NPI score (informant-reported neuropsychiatric symptoms) in our subjects. BIS positively correlated with GDS score (*r* = 0.21, *P* = 0.007), and BAS negatively correlated with GDS score (*r* = −0.20, *P* = 0.007), suggesting higher self-reported depression predicts greater observer-reported behavioral inhibition and lower levels of behavioral activation. There is no significant correlation between BIS/BAS score and NPI total score. However, BIS positively correlated with NPI depression subscore (*r* = 0.40, *P* < 0.001), negatively correlated with NPI disinhibition subscore (*r* = −0.22, *P* = 0.036), positively correlated with NPI irritability subscore (*r* = 0.22, *P* = 0.004), and BAS positively correlated with NPI agitation subscore (*r* = 0.21, *P* = 0.05). NPI apathy subscore did not correlate with BIS or BAS scores.

## Discussion

### Summary of the results

This study revealed that the BIS/BAS questionnaire was able to depict different patterns of motivational set among patients with bvFTD, rtFTD, svPPA, and AD, and suggests that the behavioral inhibition system-related behaviors may have correlates in the brain structure of these patients. In particular, a behavioral tendency toward higher levels of anxiety in the face of punishment cues, and the corresponding avoidant motivational set, appears to correlate with volume in predominantly right frontal regions including the right anterior cingulate, right precentral gyrus, and right middle frontal gyrus, a result which appears across the diagnostic groups represented in our sample, and thus represents a brain–behavior relationship generalizable beyond neurodegenerative disease. This is consistent with previous literature demonstrating a relationship between dorsomedial/dorsolateral right frontal damage and loss of social inhibition (Gray and Braver 2002; Asahi et al. 2004) (Eisenberger et al. 2005). It also suggests that loss of self-conscious behavior may be a direct result of damage to circuits underlying the BIS system. While our sample was underpowered to depict structural regions significantly predicting activation system behaviors, we found that patients in different diagnostic groups demonstrated divergent behavior across subscales of the BAS. We also found evidence at a statistically subthreshold level that both drive and fun-seeking subscales show predictable correspondence to dorsomedial frontal anatomy, while reward responsiveness corresponds to ventromedial subgenual frontal regions known to mediate reward-related evaluation.

### Neuroanatomy underlying the BIS and BAS systems

The fact that our study found a relationship between the BIS system and volume change in predominantly right-hemisphere structures is consistent with theories that emphasize the role of the right hemisphere in emotion (Silberman and Weingartner 1986), and specifically the right frontal cortex in behavior inhibition during emotionally charged or conflict situations. In their recent review article, Kennis et al. (2013) hypothesized that BIS-related personality traits are positively correlated with activity in an amygdala-hippocampus-PCC network (PAG-medial hypothalamus-amygdala-hippocampus-PCC-dorsal PFC) that activates in response to conflict. BIS may predict individual differences in the capacity to focus sustained attention on environmental cues during conflict, particularly when they are related to potential reward or punishment. For instance, Gray et al. (2005) evaluated sustained neural activity in regions associated with cognitive control during a demanding working memory (3-back) task. Individuals with higher BIS scores show significantly greater sustained activity in the same rostral anterior cingulate cortex (ACC) region found in our study, supporting its role in active, sustained inhibition during cognitive conflict.

Some of the cortical regions corresponding to BIS in our study overlap with structures seen in the stable task-control network (also termed the sustained attention network) (Dosenbach et al. 2007; Seeley et al. 2007), that has been described as a result of recent attempts to delineate the intrinsically connected resting-state networks (ICNs) that underpin complex cognition. The hypothesized function of this network is to maintain an “on” state during tasks in a manner that underpins attention, even during divided attention tasks. While previously understood in the context of task activity, the overlap of this network with behavioral inhibition functioning suggests it might play a role in the temporally extended maintenance of personality traits related to self-control, self-concern, avoidance of errors, and social inhibition. Components of this task-control network seen to correlate with BIS in our study include the ACC, which plays a role in error monitoring and top-down maintenance of task set (Dosenbach et al. 2007). Importantly, subregions of the ACC are shared across multiple ICNs, and the lack of anatomic precision involved in the structural VBM method used in our study suggests we take a conservative approach in hypothesizing the function of this region in BIS responsiveness.

The ACC also is tightly linked with the anterior insula in the salience network and with emotion generators including the amygdala and hypothalamus, thus is hypothesized to be essential for survival-relevant affective stimuli detection and visceromotor emotion generation (Seeley et al. 2007). The BIS system is associated with negative, inhibitory affect, thus the role of the ACC in this system may be to generate negative emotion in response to potential punishment cues. There is also evidence that high BIS individuals may even process reward differently. During a monetary incentive delay task, subjects with a high BIS score show less activation in the ventral striatum during the receipt of a reward (Simon et al. 2010). This combination of negative emotion generation and reduced reward responsiveness may serve to enhance the high BIS individual’s tendency to avoid reward-related situations.

The right middle frontal gyrus, another region found in our study that is part of the sustained task-control ICN, is believed to be part of a complex network mediating emotion-driven influences on action selection, potentially mediating working memory functions involving socioemotional material (Bechara et al. 2000; Rolls 2000). Volume loss in right middle frontal gyrus is associated with less inhibition in patients with bipolar disorder (Haldane et al. 2008), and predicts frequency of errors made by bvFTD patients during testing (Possin et al. 2009).

Other regions found in our study to directly correlate with BIS score may be involved more directly in emotion regulation. The right inferior frontal cortex region found in our study, which is also a part of the task-control ICN, has been shown to be involved in emotion regulation, which is likely an important function of the BIS system (Spunt and Lieberman 2012). A recent functional MRI study of emotional control found greater activation in the right precentral gyrus during emotion regulation (Seo et al. 2013).

Despite these brain–behavior correlations found in the BIS system, we did not find any significant correlations between any BAS score and gray matter volume. We speculate that this might have occurred because the behavioral activation system is determined not only by neural structures but also other functions such as neurotransmitter function. For example, dopamine functioning plays a significant role in both motivation and behavioral activation (Salamone and Correa 2012). Next, there is a possibility that the range of BAS subscores was so restricted in our sample that the scores did not correlate significantly with cortical volume of neurodegenerative disease. The BAS-D and the BAS-FS ranges from 0 to 20, but the mean is around eight and the standard deviation is around two. These ranges are much narrower than the BIS (range 0–35, mean is around 16–17 and standard deviation is around 3.5). Also, the effect size in the brain–behavioral correlations in BAS may have been modest, and because our sample was fairly small for a covariates-only VBM analysis, resulting in a reduction in power. We did find some neurologically meaningful relationships at an uncorrected threshold, which might be replicated in other studies better powered to detect these relationships. Each BAS subscale had an anatomically distinct correlate, arguing that it may not be useful to perform brain–behavior analyses using the total BAS score. The dorsal anterior cingulate is well established as a structure underpinning behavioral drive (Gasquoine 2013), thus its subthreshold association with the BAS-drive score is likely genuine. Similarly, the subgenual cingulate areas corresponding to BAS-reward responsiveness are known to be central to reward processing (Lallement et al. 2014). Less predictable was the association between the dosomedial frontal gyrus and BAS-Fun-Seeking score, though again the dorsomedial frontal cortex is responsible for many aspects of behavioral activation, thus the motivation toward positive stimuli might also be partly mediated by this region.

### Divergent patterns of inhibition/activation across patient groups

Accurate diagnostic discrimination of bvFTD from AD is important because the qualitatively different socioemotional symptoms of bvFTD have profound implications for both patients and their caregivers (Riedijk et al. 2006; Diehl-Schmid et al. 2013). Inaccurate diagnosis may cause delayed, inappropriate treatment and families may be subject to increased distress. Studies repeatedly suggest that neurobehavioral assessment seems to be more sensitive than traditional cognitive testing for diagnostic discrimination of bvFTD from AD (Hutchinson and Mathias 2007; Mathias and Morphett 2010). In our study, patients with AD showed significantly higher BIS score than NCs, suggesting they have abnormally high sensitivity to negative cues, such as signals carrying threat of social punishment or fear stimuli. Examination of item responses in AD patients suggests higher BIS scores are associated with worrying, rumination, and a sense of possible danger or loss. In our sample, depression score (GDS) did not differ significantly between AD and the other patient groups, so this increased behavioral inhibition cannot be fully explained by the presence of depression. AD patients also showed lower BAS-Fun Seeking score than NC groups, suggesting reduced desire for new rewards and low willingness to approach a potentially rewarding event. Zelenski et al. found that BAS-FS is highly correlated with an impulsivity dimension (Zelenski and Larsen 1999) and Smits et al. reported that neuroticism is highly correlated with the combination of high BIS and low BAS-FS (Smits and Boeck 2006). In this sense, our results are consistent with previous studies of personality change in AD patients reporting decreases in extraversion/increases in introversion, and an increase in personal distress and neurotic tendency (Duchek et al. 2007; Sollberger et al. 2009; Sturm et al. 2013). Several researches have been shown that AD patients have hyperactivity of the intrinsically connected salience network involved in attention to survival-relevant-stimuli (Zhou et al. 2010). Our data suggest that these symptoms in patients with AD may be better described as a combination of hyperactivity of the behavioral inhibition system, that is, increased attention toward punishment and fear cues, coupled with reduced tendency to approach potentially rewarding events. Anatomically, patients with heightened behavioral inhibition were more likely to have preservation of right-sided structures in the dorsal (task control) aspects of the salience network, suggesting preserved and even abnormally high function in this network might be responsible for this AD-specific behavioral profile.

Patients with bvFTD showed significantly lower reward responsiveness (BAS-RR score) than NC and AD groups. Higher BAS-RR scores predict positive affective responses to the signals of impending reward (Carver and White 1994). This may suggest that asking for family reports on the 5 BAS-RR items provides diagnostic discrimination from AD and may be a more specific, psychometrically valid alternative to more lengthy neuropsychiatric measures of apathy.

This reduced reward responsiveness in bvFTD patients is consistent with the finding that they show more pronounced apathy than AD patients (Chow et al. 2009; Eslinger et al. 2012), to the degree that it is part of the diagnostic criteria for bvFTD (Rascovsky et al. 2011). Apathy in bvFTD is also known to have a distinct clinical profile characterized by loss of interest in personal affairs and responsibilities, social withdrawal and loss of awareness of personal hygiene. (Quaranta et al. 2012), related specifically with prominent atrophy in the ventral striatum (including the right caudate) (Eslinger et al. 2012). This phenomenologic difference in apathetic behavior between AD and bvFTD may arise from differences in the BAS motivation regulation system; our results suggest that the apathy in bvFTD patients may be partly characterized as reduced function in the reward responsiveness aspect of behavioral activation system.

Unlike all of the other dementia groups, whose level of behavioral inhibition (BIS score) was quantitatively elevated, bvFTDs remained normal compared to NCs. While high BIS was related to preservation of right-predominant dorsal task-control/salience network structures, which appear to enable self-protective avoidance of potentially negative stimuli, damage to these right frontal regions and amygdala is pathognomonic to bvFTD and may explain why these patients lack self-consciousness despite their regular episodes of rudeness in social settings. The fact that BIS score was not statistically lower in bvFTDs than controls may actually be the result of the genetic and pathological heterogeneity among bvFTD patients, supported by the fact that the standard deviation of BIS score in the bvFTD group was larger than in the other groups. bvFTD patients have diverse patterns of right frontal damage (Whitwell et al. 2009), thus the severity of related behavioral features will be variable. For instance, some individuals carrying the C9ORF72 mutation are reported to experience higher anxiety, at least at early disease stages, thus may have some preservation of the behavioral inhibition system, allowing them to continue to experience self-conscious anxiety and fear of errors where other bvFTD patients do not (Sha et al. 2012).

This hypothesis is supported by the quantitative elevation in BIS scores in rtFTDs, who presumably have less right frontal damage compared to bvFTDs (Whitwell et al. 2012). However, potentially due to the small sample size of these patients, these higher BIS scores did not reach statistical significance. A subset of right temporal FTD patients are reported to show intense and even obsessive self-concern, such as concern about their illness or about their functional capacity (Seeley et al. 2005), and have more accurate self-awareness than typical bvFTDs (Shany-Ur et al. 2014), although there is a possibility that angnosia may have effect. rtFTD patients also showed abnormally low fun-seeking behavior (BAS-FS score), indicating reduced desire for new rewards and unwillingness to approach a potentially rewarding event. rtFTD patients also often develop narrowed preoccupations, with emotional blunting and a flat or bizarre affect, marked loss of empathy and interest in others, rigidity, and compulsivity, (Mychack et al. 2001; Thompson et al. 2003; Liu et al. 2004), symptoms which often precede cognitive findings by years (Seeley et al. 2005). Our results suggest that dysfunction in the behavioral activation system that includes a lack of desire to approach potentially rewarding events may be responsible for some of these social and behavioral symptoms. Potentially, if right temporal patients do experience some loss of capacity to invest meaning in complex or novel stimuli due to subclinical semantic loss, they may fail to correctly judge the potential for reward in novel events, experiences, or people. However, our structural VBM results did not provide information about the mechanism underpinning this constellation of behavior in rtFTDs, as there was not a significant relationship between brain volume and BAS-FS score.

Patients diagnosed with svPPA showed no significant difference from other groups on any of the BIS or BAS scales, suggesting no clinically meaningful changes in their approach or withdrawal motivation in a social setting. While several previous studies described emotional and behavioral changes in svPPA patients, (Snowden et al. 2001; Rosen et al. 2006) these studies typically include patients with predominantly right-temporal disease along with more typical left dominant patients. In our study, we divided temporal patients into two groups (svPPA and rtFTD), thus patients with predominantly right temporal damage were not included in our svPPA cohort, which may have reduced the prevalence of more dramatic behavioral features in this group. However, even svPPA patients selected in this way demonstrate altered capacity to understand insincere communication and other complex social signals (Shany-Ur and Rankin 2011). Thus, our results suggest that while the pattern of altered social cognition in svPPA patients is likely complex and multifactorial, it may not occur as a result of deregulation of approach or inhibitory motivational set or reward-related behavior.

### BIS/BAS as predictors of neuropsychiatric symptoms

There were weak but significant correlations between higher BIS and lower BAS scores with increasing depression (GDS score) in our sample. This is consistent with previous studies showing that depressed patients have lower BAS levels and higher BIS levels (Kasch et al. 2002). While overall severity of neuropsychiatric symptoms (NPI total score) did not correlate with BIS or BAS score, higher levels of behavioral inhibition predicted higher levels of depression and irritability, and lower levels of disinhibition among the patient groups. Behavioral activation (BAS-total score), on the other hand, predicted higher levels of agitation on the NPI. This is an indication that preserved drive and reward responsiveness may actually be a potential contributor to what family members perceive as agitated behavior in neurodegenerative disease patients.

### Effect of normal aging on BIS/BAS score

In our very carefully characterized cohort of neurologically healthy older adults (age 46–90), there was no significant relationship between age and BIS or BAS score, suggesting the behavioral inhibition and activation systems may be independent from healthy aging effects. Previous studies with less well-characterized community samples representing a wide range of ages (age 18–79) reported that both BIS and BAS scores were lower in older groups than in younger individuals (Jorm et al. 1999). However, the average scores and ranges between their cohort and our cohort are largely different (mean BIS score in their cohort is around 20, while that in our cohort is around 16). Cultural issue and sampling method (community samples potentially include disease subjects vs. highly educated individuals who volunteered for a clinical research) may have some effect on this difference. Thus, we believe that behavioral motivations systems may plateau in middle age.

### Limitation

There are some methodological issues that should be mentioned in order to understand these results fully. First, because the VBM method is based on an atrophy model that relies on the use of subjects with diverse atrophy patterns, the extent to which results can be generalized beyond a study’s population is an issue of debate. However, this method has been used to accurately localize cognitive functions to brain areas in healthy controls using other, nonatrophy-based techniques (Amici et al. 2007), suggesting that generalization is possible and appropriate. Nevertheless, the influence of disease specific patterns of coatrophy remains a potential confound. Thus, we performed two additional analyses designed as error checks, which increased the likelihood that our results are not restricted to our study sample but are generalizable to normal brain function. Second, the degree to which structural VBM is truly a whole-brain analysis is limited by the particular composition of the subject sample. Our study included patients with diseases known collectively to affect most cortical structures in order to maximize sample-wide variability in both brain atrophy and behavior. However, it remains possible that some brain regions might have suffered from restriction of range and a corresponding loss of power to detect brain–behavior relationships, particularly in cases where only small numbers of subjects had atrophy to an important region. Additionally, our cohort sample sizes were relatively small, especially in temporal variant groups. Thus, there is a possibility that medium to small effect sizes might not have been detected statistically. In a related caveat, our sample of healthy older controls was statistically older than our patients, though this was added as a covariate to all analyses. Another consideration is that an informant version of the BIS/BAS scale was used, as patients with neurodegenerative disease often do not have insight into their behavioral problems, and may provide inaccurate answers on self-report measures. Though informant-based measures also may be biased, particularly when the caregiver’s own well-being is compromised, this is preferable to self-report, and this informant-based questionnaire approach is widely used with neurodegenerative patients. Another methodologic caveat is that participants underwent one of three different types of MRI scans; however, we included scanner type as a covariate in all analyses. Finally, the range of BIS and BAS scores (especially BAS subscore) in our sample was relatively small, meaning that while statistically significant differences were seen in patients at the group level and these differences correlated with structural anatomy, distinguishing clinically significant differences in individual patients may be more difficult. However, the nearly 4-point distinction between typical AD and bvFTD patients suggests this may remain useful for differential diagnosis.

## Conclusion

Our study revealed that when neurodegenerative disease patients show a behavioral tendency toward higher sensitivity to punishment cues and the corresponding avoidant motivational set, this behavior correlates with preservation of volume in right-predominant dorsal task-control/salience network regions including the right anterior cingulate, right precentral gyrus, and right middle frontal gyrus. The overlap of this network with behavioral inhibition suggests that it might play a role in the temporally extended maintenance of personality traits related to self-concern, avoidance of errors, and social inhibition. We also found that behavioral activation patterns high in drive and fun-seeking show predictable correspondence to dorsomedial frontal anatomy, while reward responsiveness corresponds to ventromedial subgenual frontal regions. On the disease level, AD patients showed higher behavioral inhibition characterized by increased sensitivity toward punishment and fear cues, together with reduced tendency to approach rewarding events. The heightened anxiety and personal distress seen in early AD might be understood in this context. On the other hand, the apathy in bvFTD patients may be partly characterized as reduced function in their reward responsiveness. Also, the damage to right frontal regions in bvFTD may explain why these patients lack self-consciousness in the context of their declining function and many social errors.

## Supporting Information

**Figure S1.** Neural substrates of BAS subscale scores.
