# Supplementary material for: Neural basis of motivational approach and withdrawal behaviors in neurodegenerative disease
Source: Brain Behav. 2015 Jul 14;5(9):e00350. doi: 10.1002/brb3.350 (PMC4589805; doi:10.1002/brb3.350)
Supplement: Supplementary file 1 [file brb30005-e00350-sd1.docx]

Supplemental Figure S1

Neural substrates of BAS subscale scores


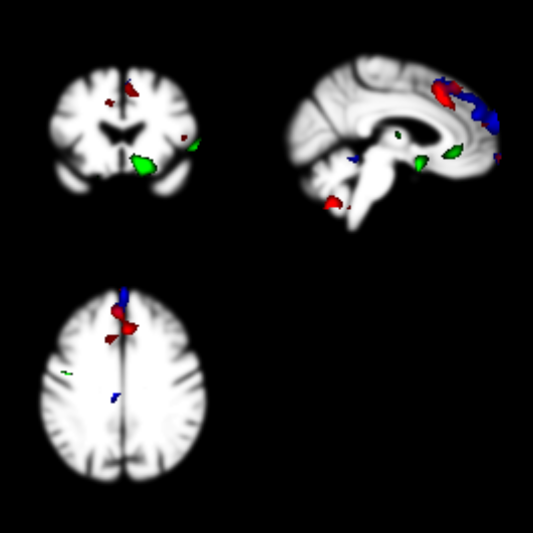


No BAS subscale score significantly predicted grey matter volume above the threshold for multiple comparisons correction (*pFWE*<0.05), nor did the overall BAS total score. Uncorrected results are shown depicting T-score maps of brain areas for which smaller volume is associated with lower drive (BAS-D score, red, max T=3.99), fun seeking (BAS-FS score, blue, max T=3.35) and reward responsiveness (BAS-RR score, green, max T=3.59). Lower threshold for t-maps T>2.00. Images were overlaid with MRIcron on an average brain based on the healthy older control gray matter template used for DARTEL warping
